# The effects of lead time and visual aids in TTO valuation: a study of the EQ-VT framework

**DOI:** 10.1007/s10198-013-0504-1

**Published:** 2013-07-31

**Authors:** Nan Luo, Minghui Li, Elly A. Stolk, Nancy J. Devlin

**Affiliations:** 1 Saw Swee Hock School of Public Health, National University of Singapore, 16 Medical Drive, Block MD3, Singapore, 117597 Singapore; 2 Pharmaceutical Health Services Research Department, University of Maryland School of Pharmacy, Baltimore, MD USA; 3 iBMG/IMTA, Erasmus University Rotterdam, Rotterdam, The Netherlands; 4 Office of Health Economics, London, UK

**Keywords:** EQ-5D-5L, EQ-VT, Time trade-off, I100

## Abstract

**Background:**

The effect of lead time in time trade-off (TTO) valuation is not well understood. The purpose of this study was to investigate the effects on health-state valuation of the length of lead time and the way the lead-time TTO task is displayed visually.

**Methods:**

Using two general population samples, we compared three lead-time TTO variants: 10 years of lead time in full health preceding 5 years of unhealthy time (standard); 5 years of lead time preceding 5 years of unhealthy time (experimental); and 10 years of lead time and 5 years of unhealthy time, presented with a visual aid to highlight the point where the lead time ends (experimental). Participants were randomized to receive one of the lead-time variants, as administered by a computer software program.

**Results:**

Health-state values generated by TTO valuation tasks using a longer lead time were slightly lower than those generated by tasks using a shorter lead time. When lead time and unhealthy time were presented with visual aids highlighting the difference between the lead time and unhealthy time, respondents spent more time considering health states with a value close to 0.

**Conclusions:**

Different lead-time time trade-off variants should be carefully studied in order to achieve the best measurement of health-state values using this new method.

## Introduction

Time trade-off (TTO) is a widely used method for eliciting health-state values. TTO determines the value of a health state by discerning how much time in full health a respondent is willing to give up in order to avoid undesirable health conditions. Despite its widespread application, TTO has been criticized because it applies different valuation procedures for states better and worse than dead. Basically, the duration of health states better than dead can be fixed (e.g., 10 years) in TTO, whereas the duration of health states worse than dead has to keep changing during the valuation process. This is the approach adopted in the Measurement and Valuation of Health (MVH) study [1]. As the respondents may engage in different psychological processes to determine indifference points in the two scenarios, the resulting negative and positive preference values may not be on the same metric. Consequently, pooling of the data is questionable [2]. Indeed, the distribution of values generated in the MVH study exhibit a ‘gap’ around 0, indicating that the values are not really continuous [3].

Lead-time TTO is a modified version of the conventional TTO [4]. By introducing a lead time into the TTO, the lead-time TTO measures all health states, either better or worse than dead, through a uniform valuation procedure. For example, a health state can be measured by asking a respondent to imagine a life of 10 years in which the first 5 are in full health (i.e., lead time) and the remaining 5 in the unhealthy state [i.e., unhealthy time (UT)] and then searching for the indifference point of the respondent between this life and *x* years of full health. With such a procedure, it is not even necessary to ask respondents whether a health state is better or worse than dead. An indifference point of less than 5 years would indicate that a health state is worse than dead and therefore has a negative value.

Lead-time TTO is a promising alternative to conventional TTO for the valuation of EQ-5D heath states, as some of those states represent very bad health conditions. Indeed, lead-time TTO was found to be a feasible and valid procedure for measuring such health states [5]. There are some caveats, however. Arguably, lead-time TTO is cognitively more complicated than conventional TTO. Further, its optimal design (for example, regarding the duration of the states to be used) is not self-evident and has to be constructed through empirical research [6]. Two previous studies have compared different lead-time TTO variants with conventional TTO. Results suggested that it is the ratio of the duration of lead time to unhealthy time, rather than the absolute duration of the two times, that affects health-state values. In one of these studies, for example, Devlin et al. [5] found that lead-time TTO variants using lead time and unhealthy time of different lengths but with the same ratio of the two times produced similar values for selected EQ-5D health states. Both of these previous studies observed an exhaustion of tradable time, which led to censored values for very bad health states, an undesirable situation also seen in conventional TTO. The two studies, however, did not agree about the effect of lead time on the valuation of health states better than dead. Values in the study of Devlin et al. were similar to or higher than conventional TTO values, while the study of Attema et al. [6] showed the opposite.

A potentially important aspect of TTO valuation is the way in which tasks are displayed. All recent lead-time TTO studies used visual aids (most commonly, TTO ‘boards’ with sliding devices to illustrate the changing length of time in the scenarios that respondents are asked to consider). In keeping with the MVH tradition, these consist of horizontal bars in different colors and lengths. Since such visual aids appeared to work well in conventional TTO, they themselves were not subjected to formal investigation. Recently, however, computer-assisted personal interview (CAPI) methods have become more common in health-state valuation studies. These have a number of potential advantages—for example, the software can display the tasks, automate the iterative process used in the TTO, and capture and time stamp all the participants’ responses. The potential use of computer software in lead-time TTO valuation of EQ-5D-5L states prompts questions about how to display the tasks and what effect the display might have on the values. To date, no information is available on how different visual aids would affect lead-time TTO valuation. In that light, their effects on valuation behavior and outcomes should be investigated as part of the search for the optimal design of lead-time TTO.

The aim of this study was to understand the effects of different lead-time TTO variants on health-state valuation. Specifically, it investigated: (1) the effect of increasing the duration of lead time from 5 to 10 years in a lead-time TTO in which the duration of unhealthy time is 5 years and (2) the effect of a special design for visual aids to highlight the length of lead time and unhealthy time on the respondents’ behavior when performing valuation tasks and on the resulting values. We expected that the findings from this study would inform the design of a software program called EuroQol Valuation Technology (EQ-VT). Developed by the EuroQol Group as a data collection system, EQ-VT elicits preference values for EQ-5D-5L health states from lay persons using lead-time TTO and discrete-choice experiment methods. The EQ-5D-5L is a new version of the EQ-5D classification system. It contains the same health domains as the EQ-5D-3L, but each domain of EQ-5D-5L has five descriptive levels [7]. The EQ-VT is intended to be the standard data collection instrument for developing country-specific value sets for the EQ-5D-5L.

## Methods

This study was conducted as one part of the pilot valuation study of the EQ-5D-5L health states, details of which are described elsewhere [8]. Briefly, this study tested the feasibility of the EQ-VT for conducting self-administered lead-time TTO valuation tasks and explored modeling strategies using data collected from eight general population samples, each from a different country. While a standard research protocol was used at all study sites to collect data for answering primary research questions, additional data were collected at four study sites, including China and Singapore, to answer some secondary research questions. Since the present study is based on data from China and Singapore only, the research designs for these two study sites are described in detail below.

### Sampling and recruitment

The study samples were generated in a similar way in China and Singapore. At both sites, information on a large cohort of the general population maintained by a commercial research company was used as the sampling frame. Members of the Singaporean cohort came from all over Singapore; all were versed in computer skills because of previous participation in on-line surveys. Members of the Chinese cohort were mainly residing in Beijing, a metropolis where many residents are immigrants from other regions of China. At both study sites, individuals were randomly selected from the sampling frames and personally invited (over the phone) to participate by a dedicated research company. Also, quotas were applied in the recruitment process at both sites to ensure that individuals of different gender and age were represented in the samples. Eligibility was assessed during recruitment and on the day of the survey against predefined inclusion criteria, notably: (1) aged 18 years or above; (2) literate and able to read from a computer screen by themselves; (3) knowing how to use a mouse and Internet Explorer to surf the Internet; (4) able to give their informed consent. In the Singapore study, literacy was defined as the ability to read English or Chinese newspapers and to converse fluently in either language.

### Procedures and instrument

Consenting participants were invited to visit a survey company where they would to complete a computer-based survey in small groups. Before starting, they were briefed by an investigator, who explained the concept of TTO and demonstrated how the EQ-VT program works. After the briefing, the group was shown to a quiet room to start the survey. Each participant was assigned a personal computer on which the EQ-VT program was started up. Participants were asked to answer the survey questions independently. Investigators were on hand to provide assistance if they encountered any technical difficulties. On completion of the survey, all participants received a monetary compensation worth approximately 15 euros. In accordance with the standard study protocol, the target sample size was 400 for both study sites.

The interview was divided into four sections. The first contained questions for warming up: participants were asked to assess their own health using the EQ-5D-5L questionnaire, give their age and gender, and answer questions regarding their personal experience with severe illness. The second section collected valuation data using the discrete-choice experiment and visual analog scale. Ten selected pairs of different EQ-5D-5L health states were presented to respondents for them to indicate preferences. Data collected in this section were not used in the present study. The third section measured the values of ten selected EQ-5D-5L health states using lead-time TTO, followed by questions seeking participants’ feedback on the lead-time TTO tasks. The fourth section gathered information about the respondents’ sociodemographic characteristics.

In the lead-time TTO section (Sect. 3), respondents were randomized to the standard or experimental arm. The TTO tasks in the standard arm were the same in the China and Singapore studies as those at the other sites participating in the multinational study. Tasks and their presentations in the experimental arm were identical to those in the standard arm except for the ratio of lead time to unhealthy time (LT/UT) in the China study and the visual display of the lead-time TTO tasks in the Singapore study.

**Fig. 1:**
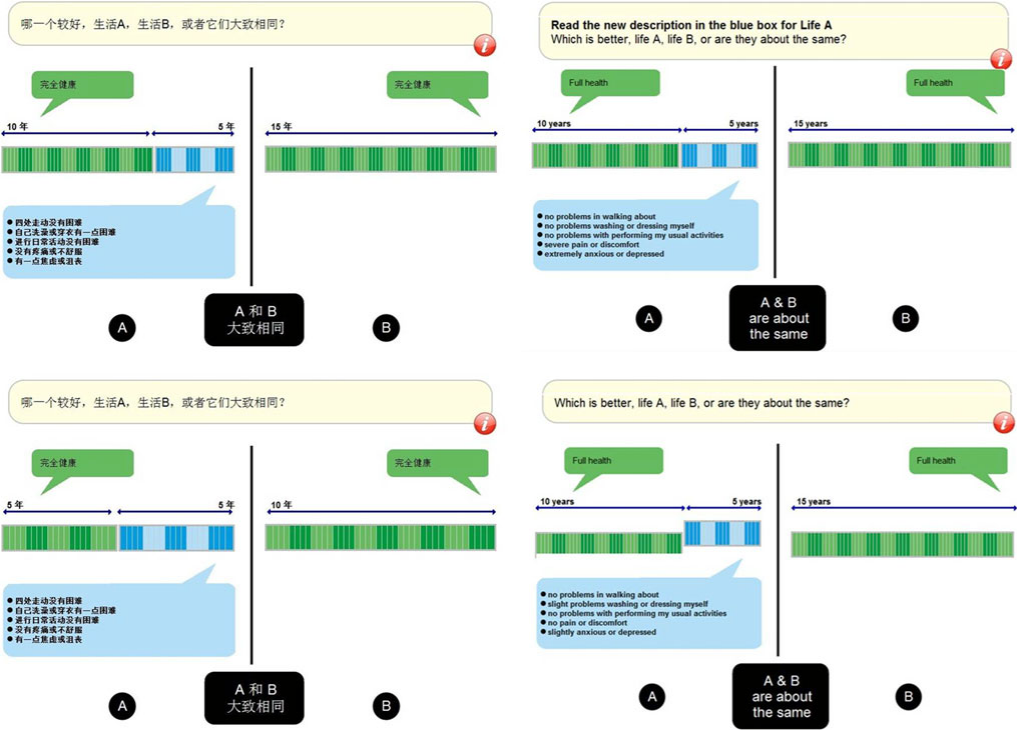
Screen shots of TTO valuation tasks presented in different study arms. *Upper left* standard arm in the China study (lead time = 10 years). *Lower left* experimental arm in the China study (lead time = 5 years). *Upper right* standard arm in the Singapore study (*bars* for time in full health and the health state being valued are aligned). *Lower right* experimental arm in the Singapore study (the *bar* for time in the health state being valued is raised)

In total, five different EQ-5D-5L health states (a subset of ten selected EQ-5D-5L health states) were presented to each participant assigned to the standard or experimental arm. The respondents had to value each state on the basis of a series of questions. Each one asked for the respondent’s preference between two hypothetical lives: living in full health for 10 years, in the state to be valued for 5 years and then die (Life A); and living in full health for *x* years and then die (Life B). Two graphs were shown side by side on the screen as visual aids to illustrate the two scenarios. The graphs contained horizontal bars; the numbers on top of the bars showed the duration of the different scenarios; the text described the health state involved (Fig. 1). Respondents were asked to click a button on the screen to indicate their answers. If the answer was Life A or B, a new question with a different *x* value would be presented and the length of the bar representing Life B would change accordingly. More questions would be asked until the answer was ‘A and B are about the same.’ In that case, the current valuation task would end and a health state with new questions would appear on the screen. How the *x* values (i.e., length of Life B) varied has been reported elsewhere [8]. Essentially, the first two values were 15 and 10 years. The third value was 12.5 (respondent 5) if the answer to the second question was Life A (or B). Values in subsequent questions changed stepwise at intervals of 3 months–1 year, depending on the respondent’s answers. The TTO tasks administered to participants in the experimental arm of the China study were identical to those in the standard arm except that the lead time was 5 years (Fig. 1). Accordingly, the number of years (*x*) posed was 10, 5, 2.5 (7.5), etc. In the experimental arm of the Singapore study, the bar representing unhealthy time in Life A was parallel to the bar for lead time but was slightly raised (Fig. 1) to highlight the distinction between time in full health and time in the unhealthy state; otherwise, visual aids in the two arms were identical.

As only a limited number of *x* values had been programmed in the EQ-VT, some respondents could not reach the indifference point for some health states. In such cases, respondents were asked to indicate indifference at the *x* value closest to his or her indifference point. For all TTO tasks, respondents were instructed to assume that the health problems they would experience in Life A will not change—for instance, through treatment—and that they would not die before the end of Life A.

### Health states

In the EQ-5D-5L system, health is defined according to five domains (i.e., mobility, self-care, usual activities, pain/discomfort, and anxiety/depression). Each domain is broken down into five functional levels ranging from no problems (level 1) to extreme problems (level 5). Each EQ-5D-5L state can be expressed using a 5-digit number. For example, 11111 and 55555 represent full heath and the worst health state, respectively. In this study, ten EQ-5D-5L states, selected to represent various severities of health problems, were divided into two blocks. Each respondent was randomized to receive one block. The health states were 12112, 52221, 33133, 44113, and 53555 in one block and 21111, 11221, 52324, 55523, and 11145 in another block.

### EQ-VT

The EQ-VT is a software package developed by the EuroQol Group for collecting valuation data for the EQ-5D-5L health states. Having been designed for computer-assisted interviewing, it runs on a server and the computers connected to it. Data collected using EQ-VT are potentially more reliable than those drawn from a conventional personal interview because they are exempt from interviewer effect and errors. EQ-VT not only provided raw valuation data, but also monitored respondents’ response style by recording details such as the number and sequence of questions and the amount of time taken to complete each TTO task. Before the start of the present study, EQ-VT was translated into Chinese by a professional translation company and then extensively tested for technical issues and understandability among the target populations. All interviews in the present study were administered on personal computers with a 14-inch monitor.

### Analysis

Data collected from the two study sites was analyzed separately using similar statistical methods. Respondents who gave all states the same value were considered not to have really understood the TTO tasks and were therefore excluded from the analysis. In addition, TTO values resulting from tasks completed in just 1 s were regarded as unreliable and also excluded. The characteristics of participants in the two arms of the study samples were compared using chi-square tests for categorical variables and two-sample t-tests for continuous variables.

The TTO value of a health state assessed in each task was calculated as *U* = (T − LT)/UT, where U is the TTO value and T is the time corresponding to the indifference point. The possible range of the value was [−2, 1] and [−1, 1] for tasks posing a lead time of 10 and 5 years, respectively. The characteristics of the TTO values and the valuation tasks were compared between the study arms using logistic regression models for categorical variables and linear regression models for continuous variables, with adjustment of age, gender, education, and health states whenever appropriate. In some analyses, the valuation observations were not entirely independent of each other because respondents contributed multiple values; in such cases, random effects were built into the model to adjust for the individual-level clustering of data.

For the China study, a simple linear regression model was used to assess the difference in aggregated TTO values between the two study arms. Those analyses were based on the mean values of the ten health states for each study arm. For the Singapore study, the number of questions answered and amount of time taken to reach the indifference point in the valuation tasks were compared between the two study arms for all valuation tasks and for tasks leading to different values. Regarding the latter, the tasks were categorized into seven groups on the basis of the values resulting from them, namely −2 ≤ *x* < −1.5, −1.5 ≤ *x* < −1, −1 ≤ *x* < −0.5, −0.5 ≤ *x* < 0, 0, 0 < *x* ≤ 0.5, and 0.5 < *x* ≤ 1. All data analyses were performed with SAS (version 9.2).

## Results

### The effect of the LT/UT ratio (China study)

A total of 406 participants completed the valuation survey in the China study. After excluding participants who gave all five health states the same TTO value (*n* = 38) and those valuation tasks completed within 1 s (*n* = 6), the 1,834 TTO values derived from 368 participants were included in the final analysis. The excluded participants were older, reported better overall health, and were less likely than those who were still included to have experienced illness among other persons (Tables 1, 2). The mean age of participants included in the final analysis was 36.1 years old. The majority of them were female (53.3 %) and had received some form of tertiary education (84.2 %). Most of them were not experiencing any problems in the EQ-5D health domains on the day of the survey, nor had they experienced illness in themselves or among family or others in the past (Table 2). A total of 194 and 174 participants completed the standard and experimental versions of the survey, respectively. No difference in demographic or health characteristics was found between the two subgroups of participants (data not shown).

**Table 1:** Demographic characteristics of participants

	China study, *N* (%)			Singapore study, *N* (%)		
	All (*N* = 406)	Included (*N* = 368)	*P* value^†^	All (*N* = 407)	Included (*N* = 379)	*P* value^†^
Gender			0.941			0.441
Male	190 (46.80)	172 (46.74)		204 (50.12)	188 (49.60)	
Female	216 (53.20)	196 (53.26)		203 (49.88)	191 (50.40)	
Age (years), mean (SD)	36.48 (10.25)	36.14 (10.19)	0.038	36.92 (11.03)	36.77 (11.04)	0.318
Education			0.097			0.074
Tertiary	338 (83.25)	310 (84.24)		53 (13.02)	46 (12.14)	
Secondary or lower	68 (16.75)	58 (15.76)		354 (86.98)	333 (87.86)	
Ethnic			NA			<0.001
Chinese	406 (100)	368 (100)		287 (70.52)	274 (72.30)	
Malay	0 (0)	0 (0)		106 (26.04)	96 (25.33)	
Indian/other	0 (0)	0 (0)		14 (3.44)	9 (2.37)	
Interview language			NA			0.995
Chinese	406 (100)	368 (100)		104 (25.55)	97 (25.59)	
English	0 (0)	0 (0)		303 (74.45)	282 (74.41)	

^†^Comparisons of included and excluded participants using Chi-square tests or Fisher’s exact tests for categorical variables and two-sample* t* tests for continuous variables

**Table 2:** Health-related characteristics of participants

	China study, *N* (%)			Singapore study, *N* (%)		
	All (*N* = 406)	Included (*N* = 368)	*P* value^†^	All (*N* = 407)	Included (*N* = 379)	*P* value^†^
Mobility			1.000			1.000
No problem	397 (97.78)	359 (97.55)		375 (92.14)	349 (92.08)	
Having problem	9 (2.22)	9 (2.45)		32 (7.86)	30 (7.92)	
Self-care			0.256			1.000
No problem	403 (99.26)	366 (99.46)		399 (98.03)	371 (97.89)	
Having problem	3 (0.74)	2 (0.54)		8 (1.91)	8 (2.11)	
Usual activity			1.000			1.000
No problem	399 (98.28)	361 (98.10)		372 (91.40)	346 (91.29)	
Having problem	7 (1.72)	7 (1.90)		35 (8.60)	33 (8.71)	
Pain/discomfort			0.145			0.647
No problem	302 (74.38)	270 (73.37)		245 (60.20)	227 (59.89)	
Having problem	104 (25.62)	98 (26.63)		162 (39.80)	152 (40.11)	
Anxiety/depression			0.286			0.110
No problem	302 (74.38)	271 (73.64)		296 (72.73)	272 (71.77)	
Having problem	104 (25.62)	97 (26.36)		111 (27.27)	107 (28.23)	
VAS, mean (SD)	89.07 (10.00)	88.81 (10.22)	0.037	84.99 (10.25)	84.87 (10.26)	0.367
Illness experience in self			0.494			0.438
Yes	25 (6.16)	24 (6.52)		29 (7.13)	26 (6.86)	
No	381 (93.84)	344 (93.48)		378 (92.87)	353 (93.14)	
Illness experience in families			0.094			0.469
Yes	135 (33.25)	127 (34.51)		157 (38.57)	148 (39.05)	
No	271 (66.75)	241 (65.49)		250 (61.43)	231 (60.95)	
Illness experience in others			0.021			0.701
Yes	93 (22.91)	90 (24.46)		132 (32.43)	122 (32.19)	
No	313 (77.09)	278 (75.54)		275 (67.57)	257 (67.81)	

^†^Comparisons of included and excluded participants using Chi-square tests or Fisher’s exact tests for categorical variables and two-sample* t* tests for continuous variables

**Fig. 2:**
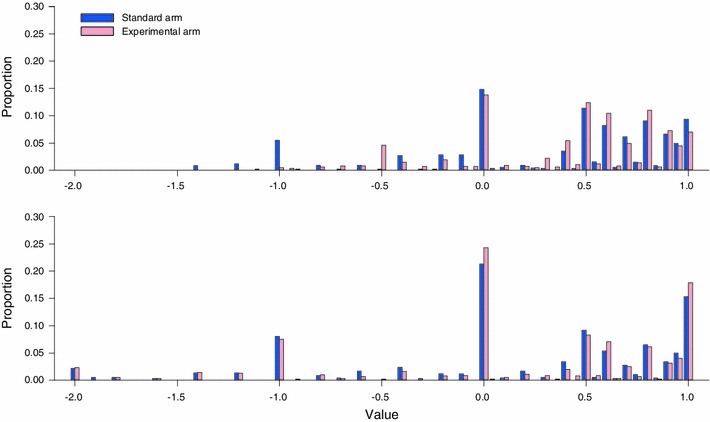
Distributions of TTO values by study arm. *Upper figure* (China study) 10 years of lead time (standard arm) versus 5 years of lead time (experimental arm). *Lower figure* (Singapore study) *bars* for time in full health and the health state being valued are aligned (standard arm) versus the *bar* for time in the health state being valued raised (experimental arm)

When all health states are considered, the TTO values derived in the two study arms exhibited similar distributions (Fig. 2). However, values from the standard arm (comprising a lead time of 10 years and a duration of 10 years in the unhealthy state; a ratio of 1:1) were lower than those from the experimental arm (comprising a lead time of 5 years and a duration of 10 years in the unhealthy state; a ratio of 0.5:1). The proportion of non-negative values in the standard and experimental arms was 81.2 and 86.7 %, respectively (*p* = 0.046); the grand mean TTO value was 0.35 and 0.43 for the standard and experimental arms, respectively (*p* = 0.049). For individual health states, the difference in values for only one of the ten health states reached statistical significance between the two study arms (Table 3). Mean values for the ten health states in the standard arm were systematically lower than those in the experimental arm. The linear model fitted by the mean values of the health states was *U*_LT=10years_ = 0.936 *U*_LT=15years_ −0.056, where the 95 % confidence interval (CI) for the coefficient is 0.567–1.306 and the 95 % CI for the constant is −0.221 to 0.018. This result suggests that the difference in values for the health states between the two arms is not significant at the aggregate level.

**Table 3:** Mean (standard deviation) TTO values by study arm

	China study, mean (SD)			Singapore study, mean (SD)		
	Standard (*N* = 964)	Experimental (*N* = 870)	*P* value	Standard (*N* = 972)	Experimental (*N* = 919)	*P* value
Better than dead, *N* (%)^†^			0.046			0.405
No	181 (18.78)	116 (13.33)		218 (22.43)	176 (19.15)	
Yes	783 (81.22)	754 (86.67)		754 (77.57)	743 (80.85)	
Health states^‡^						
11145	0.32 (0.60)	0.41 (0.42)	0.279	0.14 (0.72)	0.04 (0.75)	0.383
11221	0.49 (0.53)	0.59 (0.41)	0.196	0.49 (0.60)	0.60 (0.49)	0.133
12112	0.50 (0.50)	0.53 (0.47)	0.554	0.57 (0.58)	0.46 (0.71)	0.233
21111	0.50 (0.56)	0.60 (0.42)	0.222	0.56 (0.63)	0.68 (0.48)	0.129
33133	0.32 (0.59)	0.43 (0.46)	0.072	0.32 (0.66)	0.36 (0.70)	0.738
44113	0.36 (0.54)	0.34 (0.46)	0.951	0.02 (0.81)	0.15 (0.74)	0.400
52221	0.38 (0.60)	0.47 (0.46)	0.193	0.08 (0.74)	0.26 (0.69)	0.151
52324	0.22 (0.66)	0.37 (0.43)	0.146	0.06 (0.74)	0.05 (0.79)	0.854
53555	0.19 (0.64)	0.34 (0.46)	0.030	−0.14 (0.84)	−0.11 (0.74)	0.841
55523	0.20 (0.63)	0.24 (0.51)	0.818	−0.13 (0.82)	−0.21 (0.81)	0.505
All health states^†^	0.35 (0.60)	0.43 (0.46)	0.049	0.20 (0.76)	0.23 (0.75)	0.667

^†^Random effect linear regression model, adjusted for age group, gender, education, and health states

^‡^Adjusted for age group, gender, and education

Exhaustion of tradable time occurred only in the experimental arm (0.46 %), where the lead time was shorter. No difference was found between the two study arms in terms of number of questions and time taken per TTO exercise (Table 4) or in self-reported difficulty in responding (data not shown).

**Table 4:** Participants’ valuation behaviors by study arm

	China study			Singapore study		
	Standard (*N* = 964)	Experimental (*N* = 870)	*P* value^†^	Standard (*N* = 972)	Experimental (*N* = 919)	*P* value^†^
Trade time exhausted, *N* (%)			NA^‡^			0.885
Yes	0 (0.00)	4 (0.46)		21 (2.16)	21 (2.29)	
No	964 (100.00)	866 (99.54)		951 (97.84)	898 (97.71)	
Number of questions used per valuation task, mean (SD)	6.62 (6.61)	6.24 (5.99)	0.302	6.16 (5.73)	7.02 (8.19)	0.055
Time taken per valuation task (minutes), mean (SD)	0.64 (0.81)	0.65 (0.81)	0.910	0.79 (0.93)	0.85 (1.00)	0.396

^†^Random effect linear regression model, adjusted for age group, gender, education, and health states

^‡^Statistical analysis not performed because of an extremely low frequency

### The effect of visual aids (Singapore study)

A total of 407 participants completed the valuation survey in the Singapore study. Twenty-eight were excluded because they gave all health states the same value. The decision to exclude them was based on the grounds that those are implausible responses. A higher proportion of Malay and Indians was excluded (Table 1). Also excluded were those TTO exercises completed within 1 s (*n* = 4), on the grounds that the data were unlikely to be reliable. A total of 1,891 observations from 379 participants were used in the final analysis. The mean age of the analytic sample was 36.8 years old, with females accounting for 50.4 %. The majority of the participants were ethnic Chinese (72.3 %), English-speaking (74.4 %), had received secondary or lower education (87.9 %), and were not experiencing any problems in EQ-5D health domains on the day of the survey and had not experienced illness in themselves, family, or others in the past (Table 2). Respectively, 195 and 184 participants completed the standard and experimental versions of the survey. The two subgroups did not differ in any demographic or health characteristics (data not shown).

No difference was found between the standard and experimental arms in terms of TTO values (Table 3), the distribution of values (Fig. 2), or self-reported difficulty in responding (data not shown). However, participants in the experimental arm (using the visual aid highlighting the distinction between the lead time and time in the unhealthy state) answered more questions and spent more time than those in the standard arm on those TTO tasks leading to values around 0 (Table 5). For example, as far as tasks leading to values in the range of 0−0.5 (excluding 0) are considered, the average number of questions (steps) used to reach indifference in the standard and experimental arms was 6.4 and 8.4, respectively (*p* = 0.152); the average time to indifference was 0.7 and 1.0 min, respectively (*p* = 0.007).

**Table 5:** Participants’ valuation behaviors in the Singapore study by resultant TTO value and study arm

Range of TTO value	Number of questions used per valuation task, mean (SD)			Time taken per valuation task (min), mean (SD)		
	Standard	Experimental	*P* value^†^	Standard	Experimental	*P* value^†^
0.5 < x ≤ 1	*N* = 394	N = 393		*N* = 394	*N* = 393	
	6.98 (6.71)	7.57 (7.76)	0.415	0.86 (1.03)	0.86 (1.03)	0.867
0 < x ≤ 0.5	*N* = 153	*N* = 127		*N* = 153	*N* = 127	
	6.37 (5.24)	8.37 (9.77)	0.152	0.73 (0.69)	1.03 (0.98)	0.007
0	*N* = 207	*N* = 223		*N* = 207	*N* = 223	
	4.62 (5.07)	5.37 (7.29)	0.466	0.72 (0.93)	0.70 (0.95)	0.692
−0.5 ≤ x < 0	*N* = 49	*N* = 32		*N* = 49	*N* = 32	
	7.55 (1.99)	12.09 (16.76)	0.108	0.84 (0.79)	1.51 (1.56)	0.038
−1 ≤ x < −0.5	*N* = 108	*N* = 87		*N* = 108	*N* = 87	
	4.31 (3.91)	4.92 (5.88)	0.881	0.67 (0.87)	0.71 (0.75)	0.547
−1.5 ≤ x < –1	*N* = 27	*N* = 27		*N* = 27	*N* = 27	
	6.00 (6.84)	5.29 (2.05)	0.546	1.01 (1.13)	0.89 (0.76)	0.551
−2 ≤ x < −1.5	*N* = 34	*N* = 30		*N* = 34	*N* = 30	
	9.06 (2.62)	8.80 (1.54)	0.100	0.95 (0.98)	0.83 (0.74)	0.094

^†^Random effect linear regression model, adjusted for age group, gender, and education

## Discussion

This study was conducted mainly to inform the further development of the EQ-VT, a standardized instrument that applies lead-time TTO to elicit health-state values. As lead-time TTO is a recently developed technique, it has not been extensively tested. Therefore, our study has important implications for both research on and the application of the TTO method.

We found that the TTO values derived from the tasks using a lead time of 10 years were lower than those using a lead time of 5 years. This result was not surprising because higher LT/UT ratios in lead-time TTO exercises were found to be associated with lower values in a previous study. Using a general population sample, Devlin et al. found that a lead-time TTO variant using a LT/UT ratio of 5 (LT + UT: 6 years) generated lower values for EQ-5D-3L health states than two other variants using a ratio of 2 (LT + UT: 30 and 15 years) [8]. However, the magnitude of differences in mean values in that study varied across health states. Differences were larger for states with severe health problems. In contrast, the difference in mean values between tasks using two different LT/UT ratios was small and almost constant across health states in our study, suggesting that similar LT/UT ratios (i.e., 1 vs. 2) would result in similar TTO values.

Several possible reasons for the effect of the LT/UT ratio on TTO values were proposed previously [6, 9]. The main reason could be the framing effect of the lead time or total tradable time used. In the present study, we think the framing effect was due to both human and design factors. People may tend to trade more time just because it is available. This is especially likely for very bad health states. Another possible human factor is visual error. In TTO valuation tasks, respondents visualize the difference in length between two lives by contrasting two bars. We know that for a certain TTO value, the higher the LT/UT ratio is, the smaller the difference in length between the two bars when indifference corresponding to that value is reached, provided that the physical length of the bars (including both lead time and unhealthy time) is fixed. Thus, at the indifference point, the difference in bars (a.k.a. the difference in length of scenarios) would seem less striking to respondents using a higher LT/UT ratio. As a result, they may tend to trade a bit more time to be able to sense the difference in bars (a.k.a. loss in life years), which leads to lower indifference points and values. This visual error is more likely to affect the valuation of states with mild health problems. The design factor is the iteration process [9], which is another potential influence on values. In this and previous studies, values for states worse than dead were searched in steps proportional to the length of unhealthy time and lead time. For example, in the present study, the next value proposed to a respondent after knowing the value of a health state is <0 in the standard (LT/UT ratio = 2) and experimental (LT/UT ratio = 1) arms was 5 and 2.5 years, respectively. Those two time points correspond to the value of −1 and −0.5, respectively, which means that respondents in the exercise using the higher LT/UT ratio were encouraged to consider more extreme values sooner and before less extreme values were tried. A recent study showed that the iteration procedure in TTO valuation affects the resulting values [10].

Therefore, as far as the two lead-time TTO variants are concerned, the one using an equal length of lead time and unhealthy time is to be slightly preferred, since a longer lead time tends to lower the produced values. This recommendation is also supported by the trivial exhaustion of tradable time occurring in the study arm using equal lead time and unhealthy time. Previous studies suggested that exhaustion of tradable time may not be completely eliminated by extending the lead time [6, 9]. One advantage of using equal lead time and unhealthy time in TTO valuation is that the transformation of negative values can be avoided, as the possible range of generated values is −1 to 1. Conventionally, negative health-state values are bounded at −1. However, it might be preferable to set both the lead time and unhealthy time at 10 years for the practical reason that it is easier to measure finer levels of values with such a time frame. For example, using 3 months as the smallest step to an unhealthy time of 10 years in the iteration process, a difference in value of 0.025 (3/120) can be detected. To reach the same fine measurement with an unhealthy time of 5 years, 1.5 months has to be used as the unit of change in the length of Life B.

In our study, we found that a special design in visual aids that was intended to highlight the time point corresponding to the value of 0 (i.e., dead) changed the respondents’ behavior during the valuation process. That is, respondents considered more values and spent more time on health states they eventually assigned a value close to 0. This result indicates that respondents thought about the values for those health states harder than those respondents who used the standard visual aids. There were two possible reasons for this phenomenon: the visual aids might be more confusing; alternatively, they might be more stimulating, prompting the respondents to reflect on their preferences more carefully. We think it was unlikely that respondents found the experimental visual aid more confusing, because they were briefed before they started and did not report extra difficulty after the exercise (Table 4). Therefore, we interpreted this result positively as an indicator of more thoughtful answers. Our hypothesis was that the special design alerted respondents whenever the TTO questions proceeded to ask for values in the proximity of dead. As a result, respondents were more likely to use dead as a term of reference, which triggered more thought and attempts of more iterations to arrive at the point of indifference. This is definitely desirable, as a major concern about the valuation elicitation procedure is the absence or inadequacy of engagement of mind. Our study therefore supports the practice of alerting the respondents when a lead-time TTO question comes to the time point equivalent to dead. It might even be useful to explicitly remind respondents that they are comparing the hypothetical life with dead when the task proceeds to that moment. However, future studies should investigate whether such a practice would lead to the ‘gap effect’ around 0. That undesirable phenomenon occurred in conventional TTO but not in lead-time TTO in this and previous studies, perhaps because dead is not explicitly mentioned during the valuation exercise [5]. Future studies should investigate whether the special design in visual aids and a physical reminder at the value of 0 would generate more accurate values.

There were several limitations to this study. First, the TTO valuation tasks were self-administered by respondents without close supervision by an interviewer. As a result, respondents might have made mistakes (e.g., failing to realize the start of a new valuation task because all health states looked very similar on the screen) or tried to take shortcuts by indicating indifference randomly. However, although this issue might impair our statistical power, we do not think it invalidates our findings. Second, time preference was not assessed or adjusted in our data analysis. It was considered as a potentially important factor in TTO, especially in lead-time TTO valuation [5]. It should thus be given due consideration in future research. Last but not least, our study subjects were Asians. Since valuation behavior may be culture-specific, we might not be able to generalize our findings to Western populations. However, no important difference in distribution of values was found between the two study samples used in this study and those at other sites of the multinational EQ-VT pilot valuation study.

## Conclusions

Our study demonstrates that different lead-time TTO variants should be carefully assessed in order to achieve the best measurement of health-state values using this new method. Respondents’ perception of the (lead-time) TTO tasks and the thought process used in responding to the tasks should be explicitly investigated in the future.
